# Mahout Perspectives on Asian Elephants and Their Living Conditions

**DOI:** 10.3390/ani9110879

**Published:** 2019-10-29

**Authors:** Hannah S. Mumby

**Affiliations:** 1 School of Biological Sciences, University of Hong Kong, Pok Fu Lam Road, Hong Kong; hsmumby@hku.hk; Tel.: +852-46435886; 2 Department of Politics and Public Administration, University of Hong Kong, Pok Fu Lam Road, Hong Kong; 3 Bull Elephant Network Project, University of Cambridge Conservation Research Initiative, David Attenborough Building, Pembroke Street, Cambridge CB2 3QZ, UK

**Keywords:** *Elephas maximus*, interview, Nepal, co-production, captivity

## Abstract

**Simple Summary:**

Mahouts, often known as elephant handlers, or by other terms, such as oozie in Myanmar, work closely with captive Asian elephants in elephant range countries. This work usually involves taking responsibility for just one elephant. The daily tasks of mahouts can include feeding, cleaning or bathing elephants, treating minor medical conditions, participating in training, and riding elephants in order for them to achieve specific tasks, such as transportation of materials, religious functions, or tourism activities. Because of their close interactions with elephants, mahouts have knowledge of elephants that stretches from the cultural to the behavioral and ecological. In this study, I interviewed mahouts in their own languages with a translator, using a mix of short and open-ended questions. The second kind of questions allowed mahouts to elaborate in as much detail as they wanted on their experience. The methods allowed me to collect perspectives on welfare and the associations with elephants in Nepal. I also asked questions about how long they had worked with elephants and how many elephants they worked with. The mahouts were not a uniform group, with differences in age, experience, and perspectives. The mahouts gave their most extensive comments when discussing elephant welfare, particularly on the use of fenced enclosures versus chains, elephant diet, and interactions with domestic animals. The input of mahouts could be useful for proposing and implementing plans for elephant welfare in range countries.

**Abstract:**

The skills, knowledge, and expertise of mahouts have been recognized by organizations and individual managers who are responsible for captive elephants and by academics, where they have been a source of studies from the ethnographic to animal behavior research. In this study, I used semi-structured interviews in local languages to explore individual experiences of mahouts in Nepal. I also investigated perspectives on elephant welfare, including the use of corral (fenced) enclosures. I undertook a further key informant interview in English to gain more discursive perspectives on the topics. Our results revealed that mahouts at the study site are unlikely to come from multi-generational families of mahouts. All mahouts referenced the religious significance of elephants in their country when describing broader local perspectives. Many mahouts explained both positive and negative implications for differing strategies in housing captive elephants, often balanced the competing interests of elephant welfare with their own need for elephants to follow verbal communication, and their responsibility for the safety of the elephants, other staff, and tourists. The fine-balancing perspectives of mahouts, taking both humans and elephants into account, underlines their role as an important source of knowledge of captive Asian elephants in range countries, and their potential role as co-producers of research linked to welfare. This approach could also be of relevance to the welfare of ex-situ Asian elephants.

## 1. Introduction

Asian elephants (*Elephas maximus*) in captivity can live in a wide range of different settings. These include, but are not limited to zoos and safari parks [1], elephant camps that are primarily for tourism activities [2], camps associated with timber extraction [3], in areas linked to transportation or wildlife observation functions, such as nature reserves [4], and in areas linked to religious and cultural functions, such as temples [5]. Although we often describe settings in terms of in-situ (within the current or historical range of wild elephants) or ex-situ (outside of this range), the enormous variation within both of those categories makes blanket statements about welfare challenging to make and almost impossible to apply. One aspect that elephants may experience across many of the captive settings is a keeper, handler, or mahout [6]. The responsibilities and activities of these individuals vary but may include feeding, cleaning, or bathing elephants, treating some minor medical conditions, participating in training, and riding elephants in order for them to achieve specific tasks, such as transportation of materials, religious functions, or tourism activities.

The term mahout is sometimes used interchangeably with elephant handlers, or with the term linked to their linguistic context, such as oozie in Myanmar [7]. Mahouts themselves often translate their title as “elephant driver”, perhaps indicating the centrality of that aspect of their occupation. The practices of mahouts vary across south and southeast Asia, but one defining characteristic is their relationship with a single elephant or small group of elephants and the associated activities linked to that individual elephant or those few individuals. The mahout system originated in Asian elephant range countries, although aspects of it have existed in ex-situ settings. For example, mahouts from the Indian sub-continent were sent to accompany elephants given as gifts to rulers in Europe [8]. Equally, there are historical examples of African elephants living in captivity and having individual handlers [9]. However, these historical interpretations of mahout-elephant interactions should be used with caution in interpreting current interactions, given the changing landscape of mahoutship [3]. Understanding current practices requires research into the practices taking place now. Given the shifting roles of mahouts in other parts of Asia, this study aims to specifically address the issues at a single camp in Nepal.

A mahout is more than just an elephant handler, as they may come from an area, a family, or a social group associated specifically with elephants and the practice of being a mahout. The context of mahoutship has been of interest to generations [10] of visitors [11], administrators and officials [7], scientists [12], and ethnographers [12,13]. These have offered interpretations of the role of mahouts both in human-wildlife interactions, and the economies and societies in which they operate. Mahout perspectives, roles, and the dynamic nature of the mahout-elephant relationship have gained increasing prominence in a range of fields including animal welfare [5] and animal behavior [14]. This study aims to specifically address mahout perspectives on enclosures, interaction with elephants, and providing the best environment for captive elephants, because these are currently important topics in Nepal, where elephant tourism is changing in response to the preferences of tourists.

In linking the perspectives of mahouts to elephant welfare, the issue of the role of what is sometimes termed “traditional knowledge” or “local knowledge” in animal welfare is raised. The terms themselves and their use are subject to debate [15], but are broadly used to describe bodies of knowledge and practices constructed, maintained, and developed by people or peoples who have histories of interaction with an environment [16]. It may form part of a complex or group of related practices, including language, naming and classification systems, land-use practices, and spiritual and religious practices [17]. Recognizing the existence of these complexes and their implications is relevant for welfare. For example, mahouts and other individuals, who work closely with elephants on a day-to-day basis, are pivotal in applying welfare practices, as they are often the ones who will implement strategies that might have been developed by managers, veterinarians, or scientists. However, their experience and skills are not only relevant in this “end-stage” of following recommendations to improve welfare. Instead, they are also a rich source of ideas, knowledge, and observations about the animals they work with and the environment they inhabit. Whilst perspectives of other stakeholders such as tourists, visitors, and other observers of mahout-elephant interactions are also pertinent in gaining human perspectives on welfare, mahouts represent an opportunity to co-produce, generate, discuss, participate in, form, test, and appraise methods linked to welfare as they are involved in the lives of the elephants on a daily basis. This avenue of research is timely, as the science of animal welfare continues to grow, and the particularly compelling case for welfare presented by elephants living in captivity is explored, including the welfare implications of their long lives [18], complex social interactions [19], and prolonged infant dependence [20].

As well as the traits of elephants as a species, in recent years the topic of individual variation and differences in elephants has come to the fore of scientific studies. This includes variation in demographic parameters such as mortality [21], age at first reproduction [22], timing of reproduction [23], hormonal profiles [23], parasite load [24], growth patterns [25], and personality [14]. Information about elephants on this fine-scale individual level has been applied to welfare, for example in developing body condition assessment frameworks and applying them at the individual level. Given the extent of individual variation in many parameters, analysis of markers within an individual at different temporal scales is often viewed as preferable to comparing across individuals at a single time point [26]. Because mahouts in particular often work with one elephant, they have been recognized as being able to provide individual-level data concerning the elephants they work with, such as on their personalities [14,27].

In this paper, I aim to address the roles and some perspectives of mahouts working with Asian elephants at a tourist camp in Nepal using interviews. First, I aimed to summarize the family history and working experience and behavior of the mahouts in terms of their interactions with elephants. This is because mahouts often come from families of mahouts, which could be a source of traditional knowledge. In some countries, the practice of long-term pairings of mahouts and elephants has broken down, and therefore I wanted to determine how this operated among the mahouts interviewed. I then investigated their perspectives on changes in the enclosures and sleeping locations of the elephants. These changes had occurred recently with the move of the camp from inside to just outside of the Chitwan National Park. Because this was an important change in the lives of the mahouts and elephants, I wanted to gain their views on how this had affected them as a case study in how mahout perspectives can be considered in such management shifts. These were open-ended questions intended for the mahouts to elaborate on the points they considered to be most pertinent. Finally, I addressed the broader perspective on mahout-elephant interactions, using a key informant interview focused on the relationship between mahouts and elephants and the characteristics of mahouts. I chose to do this because the semi-structured interviews restricted what this individual wanted to address, and as he was fluent in English and wished to discuss more, I took the opportunity to gain this wider perspective.

## 2. Materials and Methods

### 2.1. Study Site

This study was conducted at the Tiger Tops Tharu Lodge (27°34′10.5″ N 84°06′06.9″ E), located just outside of Chitwan National Park, Nepal in January 2019 over 10 days. The Lodge’s primary function is for-profit wildlife tourism. It also engages in philanthropy as part of its wider activities. The mahouts at the lodge are from the local Tharu communities. Their working conditions and economical security may be higher than that of mahouts at other camps because of the “high-end” nature of their workplace. Ten female Asian elephants were housed by the Lodge, each in a fenced enclosure known as a corral. They were in the corral alone (*n* = 4), but within visual, auditory, and olfactory contact of another elephant or elephants, or in a shared corral with one other elephant (*n* = 6). In total, the corrals cover 15 acres. All elephants overnight in the corrals. They also feed in their corrals.

Each elephant had a first mahout, who performed the primary mahout roles including “driving” the elephant, directing it using foot movements and verbal signals whilst sitting on its neck. This riding activity was only done by mahouts mahouts, including during grass cutting and walks in the forest. No rides for tourists were offered. The elephants were trained with tools such as hooks and sticks, most of them in India before they joined the camp. They are primarily directed with only verbal commands and feet, when the mahouts are riding. However, the mahouts do carry sticks to direct the elephants in case they come across wild animals on forest walks. Activities included feeding the elephant, bathing the elephant, and other activities as described by the mahouts in the Results section. The second mahout of elephants performed these duties when the first mahout was on leave, and further he (all mahouts identified as male) also prepared food including bundling grasses into bales, making kuchis or “elephant sandwiches”, a mix of chickpeas, molasses, and rock salt wrapped with paddy. These wraps are given to elephants as a supplement to their primary diet. The second and first mahouts also clean the corral areas and remove dung to be utilized as fertilizer. The elephants might be engaged in tourism-related activities for up to two blocks of 2–3 h per day.

The corral system was introduced in 2016, after the elephants were moved from being kept in the National Park to just outside of it. Before the move, they were kept on a chain (1.5 m long) overnight. It was attached to one leg and the elephants were provided with a cover over this area to protect them from dew and rain. They spent time foraging in the surrounding forest areas in the day. They were involved in the same suite of tourism-related activities, which then also included tourist riding. The change to the system of corral living, made as a welfare decision to improve the living conditions of the elephants, marks a significant transition in the lives of the elephants and the working lives of the mahouts.

### 2.2. Data Collection

Interview tools are often used in cases in which answers to open-ended questions are solicited. They are increasingly used in ecological and environmental sciences in order to obtain information about attitudes, behaviors, and preferences, amongst others [28]. First, I collected summary data on all 10 of the mahouts in the camp using a semi-structured interview tool. This tool was approved by the University of Hong Kong Human Ethics Research Council (EA1903021). Since first and second mahouts often organize leave around each other, these represented all the mahouts in the camp and comprised nine first mahouts and one second mahout, one for each elephant in the camp. The interview method was chosen to allow mahouts to elaborate on their views and to avoid designing categories which they have to modify their responses to fit around. The questions included the history of mahoutship in the mahout’s family, duration of employment as a mahout, the number of elephants worked with, and short-answer questions on the birth, origin, health history, and reproductive history of the elephant. Each mahout was explicitly asked about the mahout-elephant relationship and perspectives of Nepali people about elephants. I then asked each of the mahouts two open-ended questions about their perspective on the elephants’ living situation in captivity to which they were allowed to respond in as much detail as they preferred. These were structured as “forced choice” questions in order to encourage mahouts to pick an option and reduce neutral answers. However, many mahouts chose to couch and balance their responses. Mahouts were asked if they wanted to contribute any further points and all declined. The complete interview protocol is attached as a (supplementary file Table S1). Interviews were conducted in person in the elephant camp, with the mahout, translator, and researcher present. Interviews lasted between 10 and 30 min and were not repeated. All the questions were asked in Nepali and translated into English by a Nepali native speaker. Interviews were audio-recorded and then transcribed in full verbatim. The translator was known to the mahouts as a colleague. The setting was informal, but the mahouts were aware of the recording, signed a consent form before the interview, and were aware of the author in the camp. For questions involving time, such as “how long have you worked with elephants?”, the numbers were tabulated.

The purpose of the interviews was to determine the information that mahouts were willing and did share in such a context, because if decisions about welfare and management are made, the mahout perspectives are solicited in such a way. Whilst the mahouts might not feel comfortable to share all their views in their workplace and with the knowledge that they are being recorded, it is the views they are willing to share in those circumstances that are most salient for contributions that can be made to welfare.

In addition to the mahout interviews, I conducted one more discursive key-informant interview with a previous head mahout and current naturalist, also employed at the site. Mahouts frame and share their knowledge and experience not in the structured interview framework, but in stories like these. That is how knowledge about being a “good mahout” is transferred. If researchers want to understand how the mahouts conceptualize mahout-elephant relationships, these stories may communicate a lot of information in a format that mahouts develop and are comfortable with themselves, rather than one formed and chosen by the researcher. The interview was conducted in English and was 1 h 35 min long. This interview also followed a protocol, but the interviewee chose to raise several points and experiences, which led to further questions in addition to the original protocol. The interview was audio-recorded and transcribed in full verbatim. All interviewees provided informed consent and were afforded the opportunity to withdraw consent at any time in line with the ethical approval for this project by the University of Hong Kong.

### 2.3. Analysis

Summary statistics, including mean and range, were calculated for the following numeric variables: time worked with the current elephant (years) and time worked with elephants in total (years). For the “yes or no” or either/or questions, the number of mahouts was totaled. These questions were: “Has your elephant had any health problems?”, “Have they had a baby (calf)?”, “Where is the elephant (elephant name) happier: in the corral or on the chain?”, and “is the elephant (elephant name) happier here (in the buffer zone) or in the jungle (national park)?” The questions were framed to avoid neutral responses. For the remaining questions, the text was analyzed to extract themes and the most common responses. These questions were: “What activities do you do with your elephant?”, “What is the personality of your elephant, what are her characteristics?”, “How does she interact with other humans?”, “How well do mahouts understand elephants?”, and “Why?” follow-ups to the questions on the elephant’s happiness in different keeping situations.

Rather than a priori making any assumptions about the responses or creating categories, the entire interview texts (English translation) were transcribed. This allowed a search for positive, such as “good”, “well”, and negative descriptors, such as “bad” and “rude”, in the text, and these were highlighted in the analysis. Searches for words associated with comparison were also used, such as “but”, “same” and so on, to analyze the balancing of responses by the mahouts. If concepts such as “respect” or “freedom” were raised in responses, they were presented. To avoid the researcher misclassifying the responses, the translation of the mahouts’ words are used and reported here as quotes, to allow the reader to consider the responses themselves.

For the more extended naturalist interview, key themes were extracted by the author from the transcribed interview in English that indicated mahout perspectives, since the interviewee had been a mahout, and his perspectives gained from training and managing mahouts. In particular, the meaning of his lived experience as he reflected on it and the descriptors he chose for mahouts and elephants were used in this analysis. In the case of “traditional knowledge” it is often through such stories and experiences that perspectives are developed, imagined, and reimagined.

## 3. Results

All 10 mahouts and one naturalist interviewed were male and all currently worked with female elephants. The mahouts reported years of experience with their current elephant as ranging from 6 months to 30 years (mean 13.2, standard deviation (S.D.) 10.5) (see Table 1). Their years of elephant experience ranged from 7 years to 44 years (mean 21.75, S.D. 12.1). Seven of the 10 mahouts said they had previously worked with elephants (outside of their training, which could account for why years of elephant experience were given as 29 for one mahout, but he stated he had worked for 28 with this elephant, and 23 years with the elephant for another mahout, but the mahout only reported 22 years of elephant experience). This experience was primarily with other elephants at the same camp. One mahout reported family members who were mahouts and no mahouts reported that their children were also mahouts.

The elephants themselves ranged in age from 13 to 58 years (based on mahout-reporting, mean 48.3, S.D. 13.1) (see Table 2). Excluding the youngest elephant, aged 13, who was born in the Tiger Tops camp to another elephant owned by the organization, all elephants originated from India. Two had unknown origins within India, the others were reported as being purchased at Sonepur (*n* = 2) and Sitamali (*n* = 5). These purchases took place around 30 years ago.

The mahouts reported the following activities as part of their routines: cleaning the enclosure, feeding the elephant, cutting grass, bathing the elephant, taking the elephant to water, trekking with the elephant in the forest, walking with their elephant in the camp, making “elephant sandwiches” (rice paddy, chick pea, and molasses parcels to be given to the elephants as treats), feeding the elephant by hand, and checking the elephants when they were sleeping. They described interacting throughout the day, with one mahout emphasizing that he thought of his elephant throughout the day, and even at night.

### 3.1. Behavior and Personality

The questions on elephant personality and interactions generated overlapping responses. Although the mahouts were not asked to frame their responses in terms of positives and negatives, eight of the 10 mahouts referred to “good” or “nice” or “bad” behavior. Examples of the positives include responsiveness to mahout directions: “She understand my verbal command, accept any verbal command…talk with her, very good understand”. Another such characteristic was not reacting to other animals: “She behave very nice. Not any dangerous animals, if any new people go in front, not dog, cow, buffalo, and she like it.” In terms of “bad” behavior, not responding to verbal cues was categories as “bad”, another example given of “bad” behavior was: “Every evening she makes a loud sound.” Behaviors associated with vocalizing to obtain food and reacting with aggression to other mahouts, visiting humans, and wild or domestic animals were viewed as negative. The questions linked to personality were mainly discussed through these specific behaviors. Many of the mahouts framed their answers as “she is good, but one thing she does is bad”, and listing those things. This suggests that the mahout position comes with an expectation of elephants following verbal signals, not reacting to other elephants, other animals, and humans, and not vocalizing to obtain food. Four of the mahouts did not give any negative examples of their elephant’s behavior. One of the mahouts characterized his elephant as “hard” to work with, but did not use any negative terms in describing her behavior. Instead, he spoke of the importance of how mahouts behave around this elephant: “The elephant give little bit sign to the mahout. At that time the mahout must be polite”. One mahout spoke about his relationship with his elephant and how that related to her behavior: “If we love each other feeding time, grass cutting time, she enjoys that.”

### 3.2. Human–Elephant Interactions

When discussing the interactions of the elephants with humans, the mahouts were primarily concerned with safety of tourists and also the ability of other mahouts to interact with their elephant. Two of the mahouts stated their elephants did not react to other humans: “Nothing any happens.” Two of the mahouts mentioned that the mahout should be present when their elephant is interacting with tourists, without being prompted to discuss safety: “Don’t do the dangerous behavior”. Three of the mahouts mentioned that new humans could arouse expectations of food provisioning, for example: “Any guest, Nepali or foreigner, and they have food, she makes a whooshing sound with the trunk. That means begging, that means, come to me, give grass, sugar cane, apple.” Six of the elephants mentioned interactions between their elephant and other mahouts. Four of these highlighted that the old mahout must be present if a new mahout is taking over: “If the new driver doesn’t understand, old driver can teach him.” Three of the mahouts mentioned that their elephant reacts negatively to other mahouts: “She becomes rude”, “she doesn’t accept”, “if goes away from mahout…If you would like to feed, only put on ground, not in mouth. From far distance.” One of the mahouts gave his answer from the elephant perspective, highlighting that the interaction was two-way: “Elephants feel this is a nice person come to my corral, give to me some food and grass.”

When discussing the understanding between mahouts and elephants, all of the mahouts expressed there was a level of understanding between elephants and mahouts. Four of the 10 mahouts expressly referred to verbal communication: “My verbal command very well understand because (I) gives love to the elephant, elephant don’t accept any command without love.” One mahout also discussed the role of “respect” in the understanding between him and his elephant. Another referred to nuance in his relationship: “They understand but sometimes is not agree.” In the context of broader perceptions of elephants in Nepal, all of the mahouts referred to the religious significance of elephants in Nepal: “Nepali people everybody say they are god.” Or: “The Nepali people say the elephant is goddess or god most have the respect this is god.” Four of the mahouts referred to specific practices that people engage in: “Most people if going to the countryside, if coming to the elephant, they pray. They say this elephant is god. If they pray, my life is getting bit longer. If I pray, I am feeling more healthy. They believe.” Or: “Sometimes try to make the red mark on the head. (Some) give to the money, food, and praying.”

### 3.3. Enclosures and Welfare

**Figure 1:**
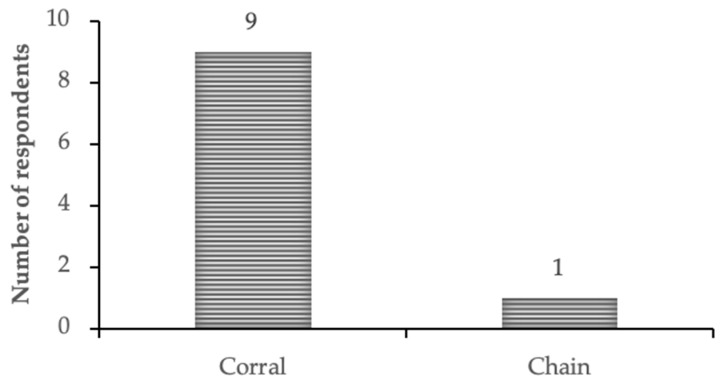
Responses to the question: “Where is the elephant (elephant name) happier: in the corral or on the chain?” Each mahout responded once referring specifically to the elephant they work with. All 10 mahouts responded. They were then able to develop their explanation further, when asked why.

The mahouts were also able to discuss a specific change that had affected their camp: the elephants had moved from the national park to the community area outside and also, they had new enclosures, fenced corrals, rather than being on chains. Nine of the 10 mahouts said their elephant was “happier” in the corral (Figure 1). Six of the mahouts specifically referred to “freedom” and ability of the elephant to move when they explained their answer: “Now is feeling very freedom when they need which place they need, if they need playing, lying down, walking they can.” Two of the mahouts specifically mentioned the ability of elephants to lie down where they chose to at night. However, four of the mahouts mentioned that mahouts interactions with elephants became more challenging with the change in enclosure, “for driver was nice when by the chain but the elephant is better than the chain inside the corral.” When asked for further information: “When I had again by the chain that time…better control for the driver. Now, something happens very difficult to the control. From time to time they get up and try to go run away, try to go left and if don’t work together at that time very difficult.” One of the mahouts noted that this situation had improved “Now is ok.” Despite these challenges and balancing the mahout needs against the elephant experience, they agreed the happiness and freedom of the elephant was most important, and that the corral enclosures were preferable.

**Figure 2:**
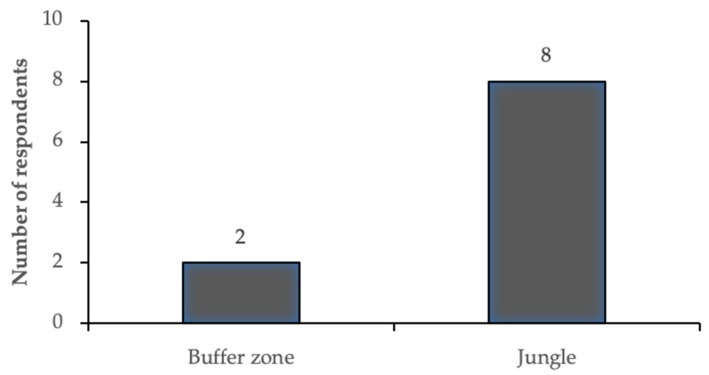
Responses to the question: “Is the elephant (elephant name) happier here or in the jungle?” Each mahout responded once referring specifically to the elephant they work with. All 10 mahouts responded. They were then able to develop their explanation further, when asked why.

In comparing the elephants living inside to outside of the national park, the mahouts also weighed positive and negative aspects of both. Eight of the 10 mahouts specified that their elephant was happier in the jungle (national park) as opposed to the current location (buffer zone) (Figure 2). Five of them expressly referred to the freedom of movement in the national park. This may seem to be in disagreement with the keeping practice, as they also referred to “freedom” when discussing corrals. However, they expressed that the elephants were able to move around all day in the park: “Everything is freedom. Only when they work that time a couple of hours controlling and after that everything is freedom.” “They’re only at night feeling on the chain and afternoon time feel the freedom in the riverbed, inside the cut line, inside the crest, there has many time, very happy.” They also emphasized the options in food choice that were available to the elephants in the park: “That time is very happy they have freedom anything is possible to eat.” Several of the mahouts mentioned the characteristics of the grasses elephants were able to select. Interviewer: “Not the same grass?” Mahout: “Not the same, different.” Finally, the mahouts pointed to the benefits of the lack of domestic animals in the park: “(Outside of the park) meet domestic cow, buffalo, dog.” Of the two mahouts who expressed their elephant was happier outside of the park, one of them mentioned the negatives of using chains and one mentioned his elephant’s reaction to wild animals: “Inside the jungle is very afraid… of the animals.” The consensus of the mahouts was that corrals were the better enclosure for the elephants. But they also expressed that being in the national park was preferable to being outside of it.

### 3.4. Mahout Stories

In addition to the semi-structured interviews, a discursive interview was undertaken with one individual who had been a mahout since 1988 and now worked as a naturalist since 2004. As with mahout interviews, he highlighted the importance of understanding between mahouts and elephants, respecting the autonomy of elephants, and that within a mahout and elephant relationship, complex interactions may take place. He did this primarily with two stories. In the first story, he spoke of being a new mahout. He had not gone with the elephant he was working with into the forest as he was a junior mahout, and his main tasks were cleaning and food preparation. The elephant was also new to the camp and had not been around wild animals before: “Ram Kali (the elephant) before I think never see rhino. Never see tiger.”

A fellow mahout decided it was time for the young mahout and Ram Kali to have their first trip into the forest. It was not long before they encountered a rhino. He describes a tense encounter: “And elephant does not understand, what is these animals? … afraided rhino also.” He noticed the fear of the elephant, whilst he and his friend sat on the elephant’s neck and back. Suddenly, the elephant reacted in response to the rhino and ran away. The more experienced mahout was thrown from her back, and to the ground, his sickle cutting through the younger mahout’s clothes. The younger mahout attempted to stay on the elephant’s neck, but he could not grip well. His only attachment was to the rope around her neck. Gradually, he slipped, until he was hanging upside down between her front legs from the rope. The elephant continued to run. “I am a hanging here… and I’m saying stop, stop.” He feared he would soon fall and die: “This is the last day.” However, the elephant used her trunk to cradle him and picked up her pace. When she slowed, he was able to free himself, but the elephant disappeared into the long grass. The young mahout was alone. He did not know whether his friend had survived the fall, particularly being so close to the rhino. However, he decided to find his elephant and search for his friend: “I’ll walking, walking and calling to my friend and we had a very far away, about five six kilometer distance, left my friend and me. And my friend walking, walking.” But the two mahouts eventually found each other, celebrating their survival: “We laughing and then we say, ok, well tonight we do need a big party plan because we have new life.” Bringing back the elephant proved to be more difficult. The elephant’s fear was palpable and she ran away every time they got close. They worked together to rejoin with the elephant: “I talking to her very polite…Elephant has the feel, my driver speak very polite.” They decided one would hold her tail and the other hold her ear and gain a foothold so he could climb up again. After several failed attempts, they succeeded. All three returned back to the camp, exhausted, but before nightfall. That night, they celebrated their safe return. He reflected on what he had learned from the experience: “Elephant and driver is very good relationship, if driver have got any problem, (the elephant) can help.”

In the second story, he describes returning from the thick forest with his elephant when the night was falling. “From the place to the tented camp one and a half hours and very dense and very dangerous place. There find wild elephant, rhino, tiger, sloth bear, leopard, very small track.” They come across a rhino and startle it: “I’m pulling the rope (tied to the elephant) and rope sound “quarrrrrrrr” and rhino is afraid they heard, my sound and go “tuh-tuh-tuh-tuh-tuh” and elephant down.” He describes falling in the commotion and injuring himself: “My nose is broken…my shirt is all tear.” He lay unconscious on the ground, “elephant feel I am not wake up and the elephant come here.” The elephant stood over him, attempting from time to time to move his body with her trunk. “I’m middle of two legs front, two legs back and I’m middle. And about my one hour after I wake up and I’m looking, plenty dark.” When he finally woke up, he was surprised and relieved to see the elephant had stayed with him as he lay bleeding and unconscious. “Really this elephant is god. Real friend, better than human. And I’m climb up, touch here, from here to here all of the blood.” He describes seeing his injuries when he returned to camp and feeling grateful to the elephant for standing by him: “Two time, I’m saying, thank God, thank elephant.”

He then explains, given his experience, what he thinks makes a good mahout. Of primary importance is a genuine interest in elephants and welfare: “Who is very interested elephant and very loving to elephant, that kind of person and he keep everything every time you have.” He said that such mahouts should be rewarded and promoted, “need a behavior very nice, very nice behave, we have him to give nice salary.” He noted that the ease with which mahouts could communicate to elephants and the responsiveness of the elephants were things he looked for when determining whether a mahout was effective. He mentioned that some mahouts might use their whole leg when riding an elephant, but a skilled mahout only needed to use the lower part. His view is that mahouts should be judged on their “nice” behavior around elephants, dovetailing with the “good” and “bad” behavior discussion that the mahouts themselves engage in regarding elephant personality.

## 4. Discussion

The study results showed some differences in the composition of the mahout community in comparison to other studies. At this site, the mahouts were not from families of mahouts. However, they did have relatively long associations with their elephants and also had extensive experience with other elephants at the site. This pattern is suggestive of a specialized group who are retained by an employer. The recent shorter relationships between elephants and younger mahouts were not observed as in Myanmar [3]. However, the site is very different, being a privately-owned high-end lodge engaged in tourism and conservation, in contrast to a government-owned commercial logging operation. The elephants, other than one, were imported and all older females, again a specific characteristic of the study site. The sample is not sufficient to generalize to other groups of mahouts at other locations but it does allow an in-depth study of a specific site. The site has notable features, including being one of the oldest lodges in the region and having a focus on “high-end” tourism. This has repercussions for the workplace stability of the mahouts. However, the study does provide a further site at which current mahout perspectives are available, which is an addition to the varied literature on the topic. For example, in comparison to a study in Nepal published in 1994 [29], the mahouts also highlighted differences in how their elephant responded to other mahouts, more slowly than to their own mahouts. The mahouts also had parallels with those interviewed in the earlier study, for example in their perception of elephant behaviors that are valuable, such as responding to cues quickly. However, the previous study suggested the mahouts were at a transition point from “traditional” families of mahouts and were increasingly viewing being a mahout as an employment opportunity [29]. This meant the mahouts had limited ability to spend a prolonged duration with one elephant. In contrast, I found that the mahouts in this study were able to work for years with one elephant. This research also highlights the value in obtaining narrative information from mahouts, as it illustrates the balance of responsibilities and perspectives they gave and highlights their specific concerns and contributions concerning welfare and behavior of elephants in captivity.

### 4.1. Behavior and Personality

Mahouts have been a source of information on elephant behavior and personality in a number of studies. Whilst I do not question their ability to contribute to studies, researchers should also be aware of their questions. I found that when mahouts are directly asked about their elephant’s personality and characteristics, the responses followed a specific format: the assumption was that all the elephants would exhibit “good” behavior, associated with compliance and responsiveness to mahout communication. Mahouts highlighted “bad” behaviors, usually linked to aggression directed at humans or conspecifics, fear of wild animals, or behavior associated with acquiring food. This pattern underlined the expectations of mahouts in terms of elephant behavior and their standards. Beyond that, it emphasizes the importance of safety and that mahouts set great store in their ability to direct and communicate with elephants. In systems where there are no physical barriers between elephants and their handlers, it is entirely understandable that this should be a priority, and has been highlighted in other studies [29]. More pertinent to researchers, it is crucial to understand that an abstract question about personality can be answered from a very concrete perspective, further emphasizing the techniques for analyzing the question and response. These could include framing a series of questions to address a single topic and evaluating the responses as a cluster rather than delineating between questions. Translation will always be an issue, both in translating questions and answers. Native speakers developing and framing questions is one strategy to ensure that multiple rounds of questions and responses are conveyed as intended, as well as the previously described strategy of question clusters. It was notable that when being a “good mahout” was discussed, communication with elephants was mentioned and the ease at which elephants responded. However, being “nice” and “polite” were also key, emphasizing that the relationship was not purely transactional. The way in which things were done was relevant as well as what was done.

### 4.2. Human–Elephant Interactions

Since mahouts occupy a unique and multi-dimensional position in terms of human-elephant relationships, it is not surprising that their answers were revealing concerning human-elephant interactions. One striking feature was the seeming division of the “sacred” and “every day” elements of the interaction. When mahouts described their relationship with elephants, they focused on their mahout role, the tasks they undertake as part of it, and contrasted their relationship with the elephant with that of the elephant with other mahouts. When they discussed how Nepali people perceive elephants, the sacred was central. It was one of the points on which the mahouts achieved a consensus. This could be because the mahouts were participating in the interviews at their place of work, and assumed that the interviewer was interested in their work. However, it still shows that the mahouts have important relationships with elephants in addition to those they perceive in Nepali people who have less contact with elephants. The only other example of mahouts having such a view came in the stories of the former mahout when he referred to the elephant saving his life. In the same story, he elevated the relationship, stating that elephants were better than humans.

The stories and reflections of the experienced mahout and naturalist spoke to these themes. They also revealed the singularity of mahout experiences and relationships. The sample size of this study was small in terms of mahout numbers, but also covered mahouts working with all elephants at the site. Moreover, the key-informant interview approach taken with one allowed more depth and expression of his personal experience in his own words, unlimited by an interview structure presented by a researcher. If researchers aim to study how concepts such as “traditional knowledge” intersect with their study systems, more open approaches should be taken to sources of such knowledge, including through stories [30]. If an informant chooses to express their views through a story, it is the story that should be analyzed, rather than restricting the construct through which they are able to respond. It is often through such stories and the recounting of lived experiences that perspectives are developed, imagined, and reimagined, and also that concepts are taught and transferred [31].

Because the elephants are managed in such an individual way, these specific associations matter as well as broad patterns and trends among mahouts. Elephants do not experience the average. Rather, they experience their specific mahouts. That the mahout and naturalist wanted to highlight formative stories and the trust he had in elephants is reflective of his mahout experience. Risks exist, but he worked with the elephant through them, and the stories had happy endings, with the mutualism between human and elephant enabling it. What is important in the stories might not be the content itself, instead how the mahout chose to present his extensive experience: in terms of cooperation, thankfulness, learning, and positivity. That as an ideal of the mahout-elephant relationship is a topic that should be the subject of further research.

Discussing the understanding between mahouts and elephants gave the mahouts some space to describe complex concepts such as “love” and “respect” and their role in the relationship. This discussion illustrated the layers of interaction between mahout and elephants that they can express in their own terms. The mahout interviews give the opportunity not just to extract quantitative information, but also allow space for reflection and consideration of the complexity on the part of the mahout. The mahouts never explicitly humanize the elephants in question, but implicitly discuss that the elephants can share love and trust with them. They show that those concepts are not limited to humans in the mahout experience. The answer from one mahout to the question “Do mahouts and elephants understand each other?” was illustrative of this, and the humor with which mahouts can express it: “They understand, but not always agree.”

### 4.3. Enclosures and Welfare

The discussion about enclosures was extremely revealing of the balancing that is part of mahout life. The consensus was that elephants were happier in corral enclosures, because of the “freedom” it gave elephants. However, the mahouts expressed they had concerns about whether elephants still followed their verbal signals with the same adherence. This presents not just a push and pull between control and freedom and how that operates for captive elephants; it also shows that mahouts do not always prefer the strategy that is easiest for them. Instead they are considering safety and welfare for all the humans and animals in the free contact system. They were in favor of enclosures that maximize an elephant’s ability to make choices for itself, as long as it still responds to the mahout. This theme is relevant to a range of captive species and methods that are used to ensure that captive animals respond to human communication within their enclosures could be relevant to mahouts, such as the target training methods often used in protected contact conditions [32].

The discussion of being in the national park versus the human-occupied zone was also a space that allowed mahouts to explore concepts of freedom and choice. In particular, food choice and variety were raised. Food is a crucial point in any captive elephant site because of the quantities required [12]. The mahouts expressed a preference for quality of food and that elephants should select their own food. This activity is often viewed as a benefit to elephants in extensive keeping systems. The comments showed both that the mahouts consider a range of factors that contribute to the welfare of the elephants in their care, and that they are balancing those elements. Whilst they wanted their elephants to experience freedom of movement and choice, they also saw limitations in the new location, including contact with domestic animals. The emphasis that elephants should be in the forest rather than around buffalo (*Bubalus bubalis*), dogs (*Canis familiaris*), and other domestic species shows nuance in the perception of the mahouts’ views of what wildness is. Although elephants are captive, their residence should be in the “wild” area with other wild animals. This perception of the best place for elephants being in the wild was also presented in an earlier study in Nepal [29]. The graded vision for the best scenario for elephants is not only in line with scientific perspectives on captive elephants—that they are not a domesticated species, rather a species in captivity—but it can also inform it, because the nature of what a wild animal is is not purely defined by their genetics, but also by their context and how the humans they live alongside define them themselves.

## 5. Conclusions

This study highlights the delicate balancing involved in mahout perspectives and the multiple roles they take in their relationship with elephants, as nurturer and feeder, protector, dependent, teacher, cooperator, and others. Mahouts have long been a source of information for a range of fields [3,6,10], which bring their philosophies, theories, and methods to interpreting the mahout role. This study shows that the semi-structured and open-ended interview methods may be an interesting tool for researchers focused on welfare and behavior and that not only short-answer, structured, or quantitative-based questions are revealing. Whilst it could be argued that mahouts answering questions about elephant behavior is more informative regarding the mahout perspective than the elephant behavior, that perspective in itself is of value given the closeness of mahout-elephant relationships.

Mahouts and others working with elephants, such as the naturalist with decades of mahout experience who gave a key informant interview, represent an opportunity for the co-production of knowledge and co-production of solutions for captive elephants. Co-production can be defined as “the collaborative process of bringing a plurality of knowledge sources and types together to address a defined problem and build an integrated or systems-oriented understanding of that problem” [33]. It is often used in the context of co-production with stakeholders in conservation [34]. This means rather than being treated as sources of information and data, potential research assistants who can acquire data, or colleagues who enact recommendations, they could potentially collaborate in addressing welfare. The interviews undertaken can generate research questions. This study showed that maintaining their ability to direct elephants in the corral keeping system and ensuring elephants have access to food choices in the forest are areas of knowledge they would benefit from research on. Mahouts highlight topics that are of interest to them, often applied and centered on their proximate challenges, such as communicating effectively with elephants. Studies on such topics are of interest both in understanding interspecific communication and also have clear application to the daily lives of mahouts.

Furthermore, the complexity of mahout answers speaks to their empathy and their continual balancing of their roles and responsibilities. Their inclusive approach to welfare, covering diffuse concepts such as “freedom” and concrete concerns such as food variety, can be used to inform our definitions and approaches to those concepts. Stakeholder participation and co-production with stakeholders are increasingly prominent topics in conservation [34,35,36], but are also clearly relevant in captive-animals studies, with mahouts being one among many examples of this. This approach can also be extended to humans working with other species and those working with elephants in different captive settings, such as zoos, logging camps, and safari parks. The wider aim of such research is making participation in welfare a common behavior and keeping proposals, decisions, and implementation more inclusive and accessible to these stakeholders.

## Figures and Tables

**Table 1:** Mahout responses to descriptive questions regarding their duration of experience and family background of mahoutship.

Years of Elephant Experience	Time Worked Together with This Elephant (Years)	From Family of Mahouts	Has Children Who Are Mahouts
20.5	14	No	No
7	10	No	No
44	10	No	No
29	28	No	No
7	1	No	No
22	23	Yes	No
24	0.5	No	No
26	8	No	No
31	30	No	No
7	7	No	No

**Table 2:** Mahout responses to descriptive questions about the elephant they work with. Locations given in India are elephant markets. Only one elephant had a known reproductive history.

Elephant Age (Years)	Place of Origin	Country of Origin	Reproductive History	Details	Health Issues	Details
56	Sitamali	India	0	NA	0	NA
13	Tiger Tops	Nepal	0	NA	0	NA
55	Sonepur	India	0	NA	1	Reproductive tract infection
52	Sitamali	India	0	NA	1	Stiff leg
50	Unknown	India	0	NA	0	NA
58	Sitamali	India	0	NA	1	Back problem
56	Unknown	India	1	Miscarriage about 14 years ago	0	NA
50	Sitamali	India	0	NA	1	Leg problem
48	Sonepur	India	0	NA	0	NA
45	Sitamali	India	0	NA	0	NA

## Supplementary Materials

The following are available online at https://www.mdpi.com/2076-2615/9/11/879/s1, Table S1: Complete list of questions asked to the mahouts.
