# Supplementary material for: Mahout Perspectives on Asian Elephants and Their Living Conditions
Source: Animals (Basel). 2019 Oct 29;9(11):879. doi: 10.3390/ani9110879 (PMC6912500; doi:10.3390/ani9110879)
Supplement: Supplementary file 1 [file animals-09-00879-s001.pdf]

animals

Supplementary files: Mahout Perspectives on Asian Elephants and Their Living Conditions

Hannah S. Mumby

Table S1. Complete list of questions asked to the mahouts.

**Mahout life information**	
1.	How long have you worked with elephants?
2.	Did you work with elephants before you worked with this one? What elephants? Where?
3.	Are you from a family of mahouts?
4.	Are your children mahouts?
5.	How well do mahouts understand elephants?
6.	What do people think about elephants in Nepal?
**Elephant information**	
1.	What is the name of your elephant?
2.	How old is she?
3.	Where does the elephant come from? (Ask where in country if only country is given)
4.	How long have you worked with that elephant?
5.	How much time do you spend together each day?
6.	What activities do you do with your elephant?
**7.**	Has the elephant had babies? When?
8.	Has the elephant had any health problems?
9.	When? How were they treated?
10.	What is the personality of the elephant? What are her characteristics?
11.	How does the elephant interact with other humans? If unsure, can give examples like guests.
**Welfare question**	
1.	Do you think (elephant name) is happier in the corral or in the jungle?
**2.**	Is (name of elephant) happier here (in the buffer zone) or in the jungle?”
